# Nanomaterials in Edible Mushroom Production: Yield Optimization, Biofortification and SDG Alignment

**DOI:** 10.1002/fsn3.71796

**Published:** 2026-05-13

**Authors:** Hayyawi W. A. Al‐juthery, Fanar Hashum Al‐Hashemi, Rukaibaa A. Chechan, Hassanein H. Al‐juthery, Heidar Meftahizade

**Affiliations:** ^1^ College of Agriculture University of Al‐Qadisiyah Al‐Qadisiyah Iraq; ^2^ College of Agriculture University of Al‐Mosul Mosul Iraq; ^3^ College of Agriculture Engineering Sciences University of Baghdad Baghdad Iraq; ^4^ Al‐Qasim Green University Babel Iraq; ^5^ Department of Horticultural Sciences, Faculty of Agriculture & Natural Resources Ardakan University Ardakan Iran

**Keywords:** biofortification, nano fertilizers, nanomaterials, sustainable development goals

## Abstract

Edible mushrooms are increasingly recognized as nutrient‐dense functional foods and integral components of sustainable circular bioeconomy systems. Nano‐enabled strategies, such as nano‐fertilizers, nano‐elicitors, and substrate amendments, significantly improve mycelial growth, nutrient uptake, and biological efficiency when applied at optimized low concentrations. Zinc oxide and selenium nanoparticles were identified as effective agents for increasing yield and enhancing micronutrient content, highlighting their role in nano‐biofortification. Postharvest applications, particularly chitosan‐based nano‐coatings and nanocomposite films, were shown to reduce enzymatic browning, microbial spoilage, and moisture loss, thereby extending shelf life and minimizing postharvest losses. At the mechanistic level, nanomaterials influence fungal physiology through controlled nutrient delivery, modulation of reactive oxygen species (ROS), enzyme activation, and improved substrate interactions. Furthermore, mushrooms contribute to sustainable nanotechnology through myco‐mediated nanoparticle synthesis and the valorization of spent mushroom substrate into biochar and other value‐added products, supporting circular economy principles. However, several challenges remain, including dose‐dependent toxicity, potential nanoparticle accumulation in edible tissues, environmental persistence and the absence of standardized application protocols. Regulatory frameworks and long‐term safety assessments are essential for responsible adoption. In conclusion, nanotechnology offers a promising pathway to enhance productivity, nutritional quality, and sustainability in mushroom production systems while contributing to SDGs 2, 3, 12, and 13. Future research should emphasize large‐scale validation, life‐cycle assessment, and the development of safe‐by‐design nanomaterials.

## Graphical abstract

Nanotechnology enhances sustainable mushroom production across pre‐ and post‐harvest stages. Nano‐fertilizers (Zn, Se) and growth stimulants (TiO_2_, ZnO) improve yield and nutritional quality. Antimicrobial nanoparticles (AgNPs) contribute to effective disease control. Nano‐enabled preservation systems (nano‐coatings, active packaging, nano sensors) extend shelf life and reduce losses. Spent mushroom substrate (SMS) is valorized into biochar and nanomaterials for reuse in cultivation. Integration of nanotechnology supports circular bioeconomy and reduces environmental impact. Precise dosing of nanomaterials is essential for safe and efficient application.

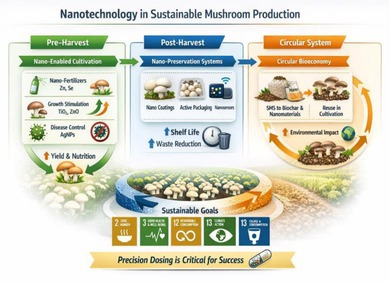

## 1 Introduction

Mushrooms that are edible and have medicinal properties are an important part of a sustainable closed‐loop system. They help create high‐quality proteins from the “waste” left over after processing lignocellulosic materials. They also produce valuable co‐products, like spent mushroom substrate (SMS). Both the transfer of SMS into other useful products and the overall process of producing mushrooms has economic effects and benefits to the rural economies (De Cianni et al. 2023; Okuda 2022). The circular economy that develops as a result of these processes supports many United Nations (UN) Sustainable Development Goals (SDGs). The specific SDGs supported are Goal 2 (Zero Hunger), Goal 3 (Good Health and Well‐Being), Goal 8 (Decent Work and Economic Growth), Goal 12 (Responsible Consumption and Production), and Goal 13 (Climate Action) (Sustainable Development Goals Knowledge Platform). There is a circular economy model based on the mushroom value chain. The production of other products, such as compost, biochar, animal feed, enzymes, and other value‐added products from SMS, can serve as an example for integrating a circular bioeconomy into the Mushroom Value Chain that will reduce waste and pollution (Grimm and Wösten 2018; Martín et al. 2023).

The application of engineered nanomaterials (characterized as having dimensions less than 100 nm) will provide cross‐cutting opportunities for improving specific productivity levels, and ultimately, enhancing and leveraging the co‐benefits associated with achieving the Sustainable Development Goals (SDGs) (Al‐Juthery et al. 2018; Al‐Moula and Al‐Hashemi 2025). For the agricultural sector, nanotechnology applications include nanotechnology‐enable fertilizers, elicitors, antimicrobial agents (including coatings) and sensors that can collectively improve the efficiency of fertilizer and other agricultural inputs by minimizing wastage when care is taken in the design of these nanotechnology enabled materials and their appropriate application (Prasad et al. 2017; Al‐juthery and Al‐Shami 2019; Balusamy et al. 2023; Al‐Hashemi and Abdaljabar 2024; Algarawi et al. 2025a). In the development of a description how myco‐nanotechnology for fungi applications has developed and is developing today with specific examples of current and future use, myco‐nanotechnology platforms will include both established and emerging uses of nanotechnology across the pre‐harvest and harvest phases of cultivating fungi through use of pre‐harvest strategies for enhancing mycelial growth, development and seed production via nanotechnology‐assisted tools in addition to other means. For example, during the pre‐harvest phase, oyster mushroom production is being facilitated through the use of small amounts (measured to be as little as 20–40 ppm) of zinc oxide nanoparticles (ZnO‐NPs) and have demonstrated positive impact on the growth of *P. djamor* and *P. florida* relative to both mycelial growth rate and yield quality. Additionally, while ZnO‐NPs were positively impacted on *P. djamor* and/or *P. florida* at those low to medium dose levels, they exhibited a reduction in positive effect for both species above those levels due to unpredictably high levels of ZnO‐NPs delivered to the mushrooms (Alzreejawi and Al‐Juthery 2020; Kumari et al. 2025).

Zinc sulfate nanoparticles (ZnO‐NPs) applied as a foliar spray at concentrations equivalent to insecticide rates have improved the total and efficient yield of mushrooms (*Pseudomonas fluorescent*) by 50% at a 10 ppm application compared to the untreated control (Shivani et al. 2021a, 2021b). The overall conclusion is that Zn can be effectively applied in the form of nanoparticles (ZnO‐NPs) to improve the total yield and efficiency of mushrooms, while contributing to the achievement of the Sustainable Development Goals (SDGs) 2, 12, and 6 (SDG's on Zero Hunger, Sustainable Responsibly Consumption and Production, Clean Water and Sanitation) by reducing the amount of zinc used per unit area and treatment duration, and that, as previous research has indicated (Kumari et al. 2025), “more is not necessarily better” and “beyond optimal concentrations of nanoparticles, the use of nanoparticles to deliver nutrients may actually suppress mycelial growth and reduce yield” implies there is a need for species/strain and substrate/dose response analyses.

The solubility of selenium and iron has been studied as potential candidates for biofortification using soluble salts; this process includes exploring the use of nano‐delivery systems to provide a safer, more bioavailable means to improve nutritional quality. The biofortification of the mushroom species *Pleurotus eryngii* with selenium by enhancing the total selenium concentration and modulating protein and polysaccharide content has shown to have healthful benefits (Ji et al. 2022a, 2022b). Furthermore, the co‐biofortification of both selenium and zinc will improve the overall quality of mushrooms of the species *Pleurotus* spp. (Madaan et al. 2024a, 2024b). There are currently numerous studies that use soluble ionic precursors to achieve biofortification; however, the use of nanocarriers may be favorable in terms of reduced rates of application and slower release time with reduced leaching, demonstrating that evidence is accumulating to support the development of sustainable nano‐agronomy (Prasad et al. 2017; Al‐Shahmani and Al‐Juthery 2021; Balusamy et al. 2023).

Nanocoating and Nanocomposite utilized for postharvest treatments can retard the fast degradation of produce caused by adverse effects of browning reactions, moisture loss, and microbial growth. For example, Chitosan‐based nano‐coatings and bio‐based nanocomposites have been found to consistently retard the loss of quality, decrease respiration and enzymatic rate of browning, and extend shelf life of produce under refrigeration. In the case of button mushrooms, a chitosan film containing nano silica particles, with possible use of the natural preservative “nisin,” has been found to decrease production of reactive oxygen species, help retain phenolics, and improve quality during refrigeration (Sami et al. 2021a). In comparison with button mushrooms, a differentiated nanometric coating composed of chitosan with hyperbranched poly‐L‐lysine has been shown to enhance and prolong “shelf life and improve quality parameters” for oyster mushrooms (Sun et al. 2024). Similar advantages for extending “shelf life and quality characteristics” may also be assumed for nanocomposites based on biodegradable nanocellulose and gelatin films containing clove essential oil nano‐inclusions (10 vol%) (Golmohammadi et al. 2024a, 2024b) or chitosan nanoparticles loaded with turmeric essential oil (3% vol) (Gong et al. 2025b, 2025a).

Process innovations that promote circularity will enable improvements to occur both before and after harvest. Fungi and *Pleurotus* sp. allow for the biosynthetic production of metallic and metal oxide nanoparticles using the extracellular enzymes produced by fungi and fungal metabolites; therefore, providing an environmentally friendly method to prepare silver, gold, titanium dioxide, and other nanocrystals without the use of toxic chemical reducing agents and intensive energy (Bhardwaj et al. 2020; Dhanjal and Cameotra 2022). As such, nano‐biocides and nanocatalysts made from fungal extract and spent mushroom substrate (SMS) filtrates can be further developed downstream and scaled locally, creating value from a typically wasted biomass component. In addition to this, SMS can be converted into micro/nano‐porous biochar by pyrolysis, which would provide adsorption and soil conditioning characteristics as well as sequester plant nutrients and reduce leaching, thereby providing monetarily positive flow paths for the largest industrial by‐product produced by the agricultural sector (Grimm and Wösten 2018; Martín et al. 2023; Algarawi et al. 2025b). Recycling and up‐cycling through these loops should significantly increase resource efficiency (SDG12), potentially reduce life‐cycle greenhouse gas emissions (SDG13), and create economic resiliency and diversity within rural economies (SDG8). The successful realization of the above benefits will depend upon careful consideration of safety, governance, and “Responsible Nano” principles at all levels.

The combination of nanotechnology and nanomaterials presents the following potential to mushroom farmers: enhance the efficiency of mushroom production with less input (nano fertilizers/elicitors prior to the harvest for yield and micronutrient supplementation, targeting SDG2), minimize post‐harvest losses with nanocoating and active packaging, targeting SDG12, and finally, work toward reducing waste in the entire value chain through myco‐synthesized nanoparticles and SMS to biochar in circular economies, targeting a combination of SDGs 8, 12, and 13. The most robust proof available has been recorded in the application of nanocoating made from chitosan and very low‐dose concentrations of zinc oxide nanoparticles; however, a dose–response strategic approach in accordance with species and strains, along with rigorous safety assessments and a life cycle assessment in the agricultural setting, will be required in the wider application of nanotechnology in mushroom production in accordance with the evolving guidelines and recommendations by the UN SDGs. This study will therefore assess how nanotechnology can be applied to improve the efficiency and sustainability of mushroom production.

## 2 Classification and Importance of Mushrooms

### 2.1 Economic Significance

Edible mushrooms hold substantial commercial value. Widely cultivated species such as *Agaricus bisporus*, *Pleurotus* spp., and *Lentinula edodes* drive global processing industries, generating billions of dollars annually (Chang and Wasser 2017). Medicinal mushrooms, including *Ganoderma lucidum* and *Cordyceps militaris*, are increasingly significant in nutraceutical and pharmaceutical sectors (Valverde et al. 2015). Specialty or gourmet mushrooms, such as *Morchella* spp. and *Tricholoma matsutake*, command high market prices due to their scarcity (Singh et al. 2022).

### 2.2 Nutritional Significance

Mushrooms are rich sources of high‐quality proteins, comprising approximately 20%–30% of dry matter, with a balanced amino acid profile (Valverde et al. 2015; Zheng, W., et al. 2025). They provide essential vitamins (B‐complex; vitamin D after UV exposure), minerals such as selenium, potassium, and iron, while being low in fat (Cheung 2010). Additionally, mushrooms contain dietary fibers and bioactive compounds like ergothioneine, which exhibit antioxidant properties (Heleno et al. 2015; Kalita, D., et al. 2025).

### 2.3 Health Relevance

Mushrooms contribute to human health via multiple mechanisms: Immune modulation: β‐glucans from *G. lucidum* and *L. edodes* enhance immune responses (Patel et al. 2019). Anticancer potential: Bioactive polysaccharides in shiitake and maitake exhibit antitumor properties. Metabolic regulation: Dietary fibers support cholesterol and glucose homeostasis (Cheung 2010). Neuroprotection: Compounds such as ergothioneine and hericenones from *Hericium erinaceus* have cognitive benefits.

### 2.4 Cultivation Methods

Mushroom cultivation varies according to species requirements:

Compost‐based cultivation: *A. bisporus* is grown on pasteurized compost derived from straw, manure, and gypsum (Chang and Wasser 2017). Log cultivation: *L. edodes* and *Auricularia* spp. are cultivated on hardwood logs under humid, shaded conditions (Philippoussis 2009). Substrate/bag cultivation: *Pleurotus* spp. thrive on sterilized agricultural residues (straw, sawdust) in bags (Patel et al. 2019). Controlled‐environment cultivation: *G. lucidum* and *C. militaris* require temperature and humidity‐controlled facilities (Singh et al. 2022; Wichaphian et al. 2025). Semi‐artificial cultivation: *Morchella* spp. are cultivated in soil‐substrate mixtures under field‐controlled conditions (Chang and Wasser 2017). Classification based on economic, nutritional, health significance, and cultivation method is summarized in Table 1.

**TABLE 1:** Classification of mushrooms according to their economic, nutritional and health importance, and cultivation method, with references.

Mushroom species	Economic importance	Nutritional and health importance	Cultivation method	References
*Agaricus bisporus* (button mushroom)	Most widely cultivated; major share in global trade	Moderate protein, B vitamins, selenium; low fat; dietary fiber	Compost‐based cultivation (straw + manure substrates under controlled conditions)	Chang and Wasser (2017); Singh et al. (2022)
*Pleurotus* spp. (oyster mushrooms)	Popular due to low‐cost production and fast growth	High protein (20%–30% DW), fiber, antioxidants; immune‐boosting polysaccharides	Substrate/bag cultivation (straw, sawdust, cotton waste)	Valverde et al. (2015); Patel et al. (2019)
*Lentinula edodes* (shiitake)	High‐value gourmet and export mushroom	Vitamin D (UV‐enhanced), lentinan (antitumor polysaccharide), essential amino acids	Log cultivation (hardwood logs) or sawdust blocks	Philippoussis (2009); Cheung (2010)
*Cordyceps militaris*	Commercially important medicinal fungus	Cordycepin and adenosine (antioxidant, anti‐inflammatory)	Solid‐state fermentation (substrate in jars)	Patel et al. (2019); Singh et al. (2022)
*Auricularia* spp. (wood ear mushrooms)	Economically important in Asia; medium‐value crop	Fiber‐rich, low fat; source of calcium and iron	Log cultivation or sawdust‐based substrates	Philippoussis (2009)
*Morchella* spp. (morels)	Very high‐value gourmet mushroom, limited cultivation	Rich in protein, iron, antioxidants	Semi‐artificial cultivation (soil–substrate mixtures under field control)	Chang and Wasser (2017)
*Hericium erinaceus* (Lion's mane)	Niche but growing market for medicinal and gourmet uses	Hericenones and erinacines (neuroprotective, cognitive health benefits)	Sawdust substrate cultivation under controlled environment	Singh et al. (2022)

## 3 Nanotechnology in Mushroom Production and Sustainability

### 3.1 Conceptual Framework

Nano‐enabled interventions enhance sustainable mushroom production through pre‐ and postharvest applications. Preharvest applications include nano‐fertilizers, nano‐elicitors, and substrate amendments, improving mycelial growth, nutrient uptake, and yield. Postharvest applications, such as nano‐coatings, antimicrobial nanocomposites, and intelligent packaging, extend shelf‐life and reduce losses. These interventions support Sustainable Development Goals (SDGs): SDG 2 (Zero Hunger): Enhanced nutrient bioavailability and productivity. SDG 12 (Responsible Consumption and Production): Reduced food waste via preservation technologies.

SDG 13 (Climate Action): Circular bioeconomy approaches, such as valorization of spent mushroom substrates (SMS) and myco‐mediated nanoparticle synthesis, increase resource efficiency and minimize waste. Risk assessment must account for environmental persistence, potential bioaccumulation, and long‐term ecological effects, ensuring responsible nano‐enabled agriculture.

### 3.2 Literature Search Strategy

A systematic literature review was conducted using Web of Science, Scopus, and Google Scholar. Keywords included “nanotechnology,” “nanomaterials,” “edible mushrooms,” “mushroom cultivation,” “biofortification,” and “postharvest preservation,” alongside sustainability‐related terms. Peer‐reviewed articles published in English between 2000 and 2025 were included. Selection criteria emphasized mechanistic insights and practical applications in mushroom cultivation.

## 4 Core Mechanisms and Applications of Nanomaterials in Fungi

### 4.1 Why Mushrooms + Nanomaterials?

Cultivated mushrooms convert low‐value lignocellulosic residues into protein‐ and nutrient‐dense food while generating co‐products (e.g., enzymes, bioactive extracts, and spent mushroom substrate, SMS) that fit neatly into circular bioeconomy models directly touching SDG 2 (Zero Hunger), SDG 3 (Good Health), SDG 8 (Decent Work), SDG 12 (Responsible Consumption and Production), and SDG 13 (Climate Action) (De Cianni et al. 2023; Grimm and Wösten 2018). Nanomaterials engineered structures typically < 100 nm add tools for precision inputs, controlled release, active packaging, and greener processing, provided their life‐cycle risks are managed (Prasad et al. 2017; Balusamy et al. 2023). As shown in the Table 2. The evidence summarized above draws on the sources listed below. Key supporting sources include Prasad et al. (2017), Grimm and Wösten (2018), Sami et al. (2021a), Ji et al. (2022a, 2022b), Bhardwaj et al. (2020), Martín et al. (2023), Matras et al. (2022).

**TABLE 2:** Integrated pre‐harvest nano‐strategies.

Application focus	Mechanism	Nano‐system	Target/process	Key outcomes	Key insight
Yield enhancement	Controlled delivery	ZnO, Fe_2_O_3_ NPs	Mycelial growth	↑ Yield, faster colonization	Low‐dose optimal
Biofortification	Ion release and transport	Nano‐Se, Nano‐Zn	Nutrient uptake	↑ Se, Zn in fruiting bodies	Improves nutrition
Growth stimulation	ROS Hormesis	TiO_2_, ZnO NPs	Stress signaling	Faster pinning	Dose‐sensitive
Substrate improvement	Water/gas regulation	Nano‐biochar, nano clays	Substrate environment	Better aeration and moisture	Improves efficiency
Disease control	Antimicrobial activity	AgNPs, CuNPs	Pathogen's suppression	Cleaner cultivation	Risk to beneficial fungi
Enzyme modulation	Surface interaction	Nano‐silica, Tio_2_	Lignocellulose breakdown	Faster substrate degradation	Enhances metabolism
Bio stimulation	Nano‐elicitors	Biopolymer NPs	Secondary metabolism	↑ Phenolic β‐glucans	Functional food value

### 4.2 Core Scientific Mechanisms Relevant to Fungi

#### 4.2.1 Mechanisms

Mass transfer and controlled delivery: Nano fertilizers release micronutrients (Zn, Se, Fe) at the hypha–substrate interface, enhancing bioavailability and reducing leaching (Prasad et al. 2017; Balusamy et al. 2023). Signal modulation and enzyme priming: Nanoparticles trigger mild ROS bursts, upregulating stress‐response pathways; optimal doses accelerate colonization, whereas overdosing inhibits growth (Prasad et al. 2017; Balusamy et al. 2023). Ion release and speciation: Metal‐oxide NPs serve as slow‐release micronutrient sources, supporting biofortification (Ji et al. 2022a, 2022b). Surface–cell interactions: NP size, shape, and surface chemistry determine adhesion to hyphae and selective antimicrobial effects (Matras et al. 2022).

#### 4.2.2 Pre‐Harvest Applications

Nano‐fertilizers and nano‐elicitors: ZnO‐NPs (~10 ppm) increased yield and biological efficiency in *Pleurotus florida* (Shivani et al. 2021a, 2021b; Qiu, Y., et al. 2021), while Se‐NPs enhanced Se content in *P. eryngii* (Ji et al. 2022a, 2022b, 2024). Substrate engineering and pathogen management: Nano‐clays, nano‐biochar, and antimicrobial surfaces modulate moisture and suppress pathogens, requiring careful selection to avoid inhibiting beneficial fungi (Matras et al. 2022) (Table 3).

**TABLE 3:** Postharvest and circular nano‐systems.

Application	Nano‐system	Mechanism	Effect on quality	Circular benefit	SDGs
Edible coatings	Chitosan + nano‐silica	Barrier + antimicrobial	↓ Browning, ↑ shelf life	Biodegradable	3, 12
Active packaging	Nanocellulose + essential oils	Controlled release	↓ Respiration, spoilage	Reduces food waste	12
Nano‐films	ZnO, TiO_2_ composites	Gas/moisture control	Maintains firmness	Replaces plastics	9, 12
Nano‐encapsulation	Essential oil NPs	Improved bioavailability	↑ Antimicrobial action	Efficient use of actives	3
Smart packaging	Nano sensors	Real‐time monitoring	Early spoilage detection	Reduces losses	9, 12
Myco‐synthesis	Fungal‐mediated NPs	Green synthesis	Eco‐friendly materials	Low‐energy production	9
SMS valorization	Biochar, nanocellulose	Waste conversion	Soil improvement	Circular economy	12, 13

#### 4.2.3 Postharvest Applications

Nano‐enabled coatings and films reduce browning, desiccation, and microbial spoilage:

*A. bisporus*: chitosan + nano‐silica + nisin films extended shelf life at 4°C (Sami et al. 2021a).

*P. ostreatus*: chitosan + hyperbranched poly‐L‐lysine coatings mitigated weight loss and enzymatic browning (Sun et al. 2024). Active packaging with essential oils (e.g., cinnamon) demonstrates synergistic preservation effects (Echegoyen and Nerín 2015; Kaur et al. 2024).

#### 4.2.4 Myco‐Routes and Circularity

Fungi can biosynthesize metallic nanoparticles via myco‐synthesis (Bhardwaj et al. 2020; Šebesta et al. 2022; Cruz et al. 2024). SMS can be converted to biochar, enhancing nutrient cycles and reducing GHG emissions (Grimm and Wösten 2018; Martín et al. 2023; Aiduang et al. 2025).

#### 4.2.5 Safety and Governance

Responsible nano‐application requires: species‐specific dose–response data, biocompatible carriers (chitosan, nanocellulose), residue monitoring, and alignment with EFSA guidance on nanomaterials in food systems (EFSA Scientific Committee 2021).

#### 4.2.6 SDG Integration

SDG 2/3: higher yield, micronutrient biofortification. SDG 12: reduced postharvest loss.

SDG 8/13: circular nano‐businesses, SMS valorization, and potential emission reductions (De Cianni et al. 2023; Okuda 2022; Martín et al. 2023). Key research priorities include standardized dose–response protocols, nano‐biofortification strategies, active‐packaging life‐cycle assessment, and EFSA‐aligned safety frameworks.

## 5 Classification and Physiological Effects of Nanomaterials

### 5.1 Classification

Nanomaterials (NMs) are increasingly explored in edible mushroom cultivation as nano‐fertilizers, bio fortifiers, elicitors and postharvest protectants. This review classifies NMs by dimensionality, composition and origin, and synthesizes evidence on their physiological effects on mushroom mycelial growth, substrate colonization, primordia formation, yield, bioactive compound accumulation, stress physiology, and postharvest quality. Benefits are typically dose‐dependent with narrow “sweet spots”; excessive dosages can inhibit mycelium or perturb the substrate microbiome. We close with practical guidance and research gaps for safe, SDG‐aligned deployment (ISO 2010, 2014; European Commission 2022; Barhoum et al. 2022).

### 5.2 Classification of Nanomaterials Relevant to Mushroom Production

By dimensionality. 0D (quantum dots, nanoparticles), 1D (nanofibers, nanotubes), 2D (nanosheets such as graphene oxide), and 3D (porous nanoarchitectures, nanocomposites) (Jeevanandam et al. 2018; ISO 2010). By composition. Carbon‐based (graphene, CNTs); inorganic (Ag, Cu, Zn; oxides like ZnO, TiO_2_, SiO_2_, Fe_3_O_4_); organic/polymeric (chitosan NPs, dendrimers); composites (e.g., nano‐silica–polymer films) (Barhoum et al. 2022; Jeevanandam et al. 2018, EFSA 2021). By origin. Natural, incidental, and engineered; EU's current definition focuses on 1–100 nm primary particles with ≥ 50% number‐based size distribution (European Commission 2022; Barhoum et al. 2022). Mushroom‐mediated (myco) synthesis. Numerous macrofungi (*Pleurotus*, *Fomitopsis*) can reduce metal precursors to metal/oxide NPs useful for greener supply chains (Bhardwaj et al. 2020; Rehman et al. 2022; Elsakhawy et al. 2022).

### 5.3 Physiological Effects in Cultivation

#### 5.3.1 Mycelial Growth, Colonization and Primordial,

Low‐dose zinc oxide nanoparticles (ZnO‐NPs) can accelerate Pleurotus colonization and morphogenesis. In *Pleurotus pulmonarius*, substrate‐amended biogenic ZnO‐NPs (∼20–40 mg kg^−1^) increased mycelial growth rate, shortened time to pinning, and improved antioxidant status (Leema et al. 2025). Nitrogen nano‐supplements (e.g., nano‐urea and amino‐rich nanoadditives such as Lithovit‐Amino25) modulate thermogenesis and N availability in *P. ostreatus* substrates, often delaying early flush but improving overall production cycle kinetics; field trials reported higher yields relative to conventional urea (Naim et al. 2021) and improved amino‐acid profiles (Sassine et al. 2021).

#### 5.3.2 Yield and Biological Efficiency

Biogenic ZnO‐NPs at ~20 ppm delivered the highest *P. pulmonarius* yields (biological efficiency up to ≈81%) versus controls (≈64%), with diminishing returns or toxicity at higher doses demonstrating a narrow optimum (Leema et al. 2025). Similar trends yielding gains with precise dosing were seen with nano‐N supplements in *P. ostreatus* (Naim et al. 2021; Poursaeid et al. 2015b).

#### 5.3.3 Nutrient Fortification and Metal Risk Mitigation

Engineered nano‐selenium added to compost reduced cadmium accumulation by ~49%–69% in *Agaricus blazei* fruiting bodies and shifted Se speciation toward organic Se (SeCys, SeMet), while modulating antioxidant enzymes, evidence that NMs can both biofortify and mitigate toxic metals through altered uptake/partitioning (Luo et al. 2025a, 2025b).

#### 5.3.4 Enzymes and Substrate Deconstruction

Nano‐elicitation frequently upregulates laccase, peroxidases, superoxide dismutase, and catalase, correlating with faster lignocellulose breakdown and primordia initiation; ZnO‐NP–induced increases in phenolics and antioxidant enzymes accompany improved growth in *Pleurotus* spp. (Leema et al. 2025).

#### 5.3.5 Disease Suppression and Competitor Control

Silver NPs (AgNPs) exhibit broad antifungal activity and have been explored as adjuncts to biocontrol for plant pathogens; formulations can suppress sclerotia and mycelial growth (Guilger et al. 2017). Claims that AgNP composites spare *Agaricus bisporus* while suppressing pathogens are promising but still preliminary and context‐dependent; in‐substrate use warrants careful residue and microbiome assessment (Guilger et al. 2017; Ameen et al. 2021a, 2021b).

#### 5.3.6 Postharvest Quality and Shelf Life

**FIGURE 1:**
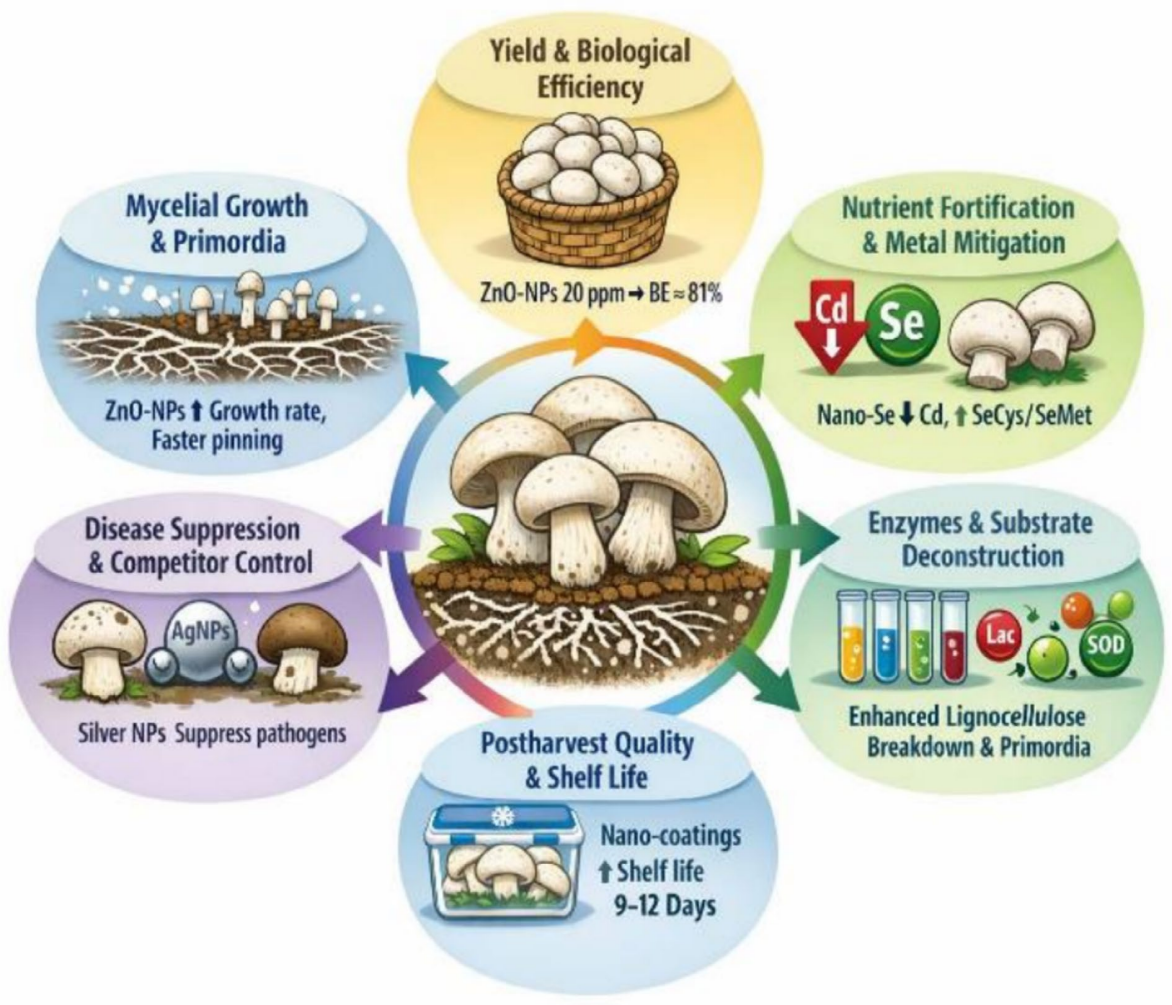
Physiological effects of nanomaterials in mushrooms.

Nano‐enabled edible coatings and films—chitosan + nano‐silica (SiO_2_‐NPs), or TiO_2_‐based nanocomposites reduce respiration, browning enzyme activity, and oxidative markers, extending refrigerated shelf life of *A. bisporus* 9–12 days versus ~3–5 days in controls (Sami et al. 2020, 2021a) (Table 4). As shown in Figure 1, which shows the physiological effect of nano materials and nano‐bio stimulants on mushrooms.

**TABLE 4:** Safety, SDG sand research priorities.

Area	Key issue	Practical guidance	Research priority	SDGs
Dose–response	Narrow optimal range	Apply minimal effective dose	Standardize thresholds	2, 3
Food safety	NP accumulation in tissues	Monitor residues and speciation	Long‐term toxicity studies	3
Environmental fate	Persistence in SMS/soil	Assess recycling pathways	Soil/ecosystem impact studies	12, 15
Regulation	Nano‐specific approval required	Follow EFSA guidelines	Harmonize global frameworks	9
Circularity	Waste reuse (SMS)	Convert to nano‐materials	LCA and sustainability metrics	12, 13
Innovation	Smart nano‐systems	Integrate sensors and IoT	Scalable technologies	9
Climate impact	Emissions and waste	Replace plastics with bio‐nano	Carbon footprint analysis	13

### 5.4 Safety and Environmental Interactions

Dose‐dependency: Excessive NP concentrations can inhibit growth or induce oxidative stress (Leema et al. 2025). Microbiome effects: Some NPs may alter beneficial fungi and substrate microbiota (Tian et al. 2019a, 2019b; Zhao et al. 2024). Regulation: EU (2022) and ISO 80004 standards define nanomaterial compliance for food systems (European Commission 2022; ISO 2010). Despite the promising benefits of nanotechnology in mushroom production, several safety and regulatory considerations must be addressed before large‐scale adoption. One important issue relates to the potential accumulation of nanoparticles within edible mushroom tissues. Because mushrooms possess efficient metal uptake systems and extensive mycelial networks, they may absorb and translocate nanoparticles from the cultivation substrate to the fruiting bodies. Although certain nanomaterials have been shown to enhance micronutrient biofortification or reduce heavy metal contamination, the long‐term implications of nanoparticle ingestion by consumers remain insufficiently understood. Another concern involves the environmental fate of nanoparticles introduced into mushroom cultivation systems. Residual nanoparticles may persist in spent mushroom substrates (SMS), which are commonly reused as soil amendments or organic fertilizers. This practice may facilitate the transfer of nanomaterials into soil ecosystems, potentially affecting microbial communities, nutrient cycling processes, and soil health. From a regulatory perspective, several international organizations, including the European Food Safety Authority (EFSA. 2021) and the United States Food and Drug Administration (FDA), emphasize the need for comprehensive risk assessments and toxicological evaluations of engineered nanomaterials used in food systems. Consequently, future research should prioritize long‐term exposure studies, environmental monitoring, and standardized safety evaluation frameworks to ensure responsible and safe implementation of nanotechnology in edible mushroom production (Fortuna, L., et al. 2023).

### 5.5 Practical Guidance for Growers and Researchers

(i) Start with agronomic NMs: ZnO‐NPs (~10–40 mg kg^−1^ substrate) or validated nano‐N supplements, titrating for species/strain and substrate; avoid “more is better.” (Leema et al. 2025; Naim et al. 2021). (ii) Prefer postharvest use for biocidal NPs: Reserve AgNPs/TiO_2_ for packaging and coatings (with migration/residue testing) rather than substrate amendments (Sami et al. 2020, 2021a). (iii) Monitor residues and nutrition: When biofortifying (e.g., Se‐NPs), quantify total and speciated elements in fruiting bodies; confirm heavy‐metal limits (Luo et al. 2025a, 2025b). (iv) Document enzyme and ROS profiles: Track SOD, CAT, POD, phenolics to understand mechanistic responses and optimize dosing (Leema et al. 2025).

### 5.6 Research Gaps

(i) Standardized dose metrics (mg kg^−1^ substrate vs. ppm in irrigation; surface area); (ii) multi‐flush productivity and quality under commercial conditions; (iii) Microbiome‐safe pathogen control strategies; (iv) Fate, transport and transformation of NMs within substrates and fruiting bodies; (v) Life‐cycle and SDG impact assessments for nano‐enabled mushroom supply chains (Barhoum et al. 2022; Tian et al. 2019a, 2019b).

## 6 Mushrooms as Healthy Foods, Their Production, and Responses to Nanomaterials

Edible mushrooms are increasingly recognized as functional foods due to their nutritional richness and bioactive compounds. They also play a central role in sustainable food systems and are being explored for nano‐enabled cultivation and preservation strategies. This review classifies mushrooms (i) as healthy foods by their nutritional and bioactive profiles, (ii) by production systems and species, and (iii) by their physiological responses to nanomaterials. References are explicitly cited by author and year throughout.

### 6.1 Mushrooms as Healthy Foods

Mushrooms are low in fat, high in protein, fiber, vitamins, and minerals, making them nutrient‐dense functional foods. According to Reis et al. (2012), widely cultivated species such as *Agaricus bisporus* (button mushroom), *Lentinula edodes* (shiitake), and *Pleurotus* spp. (oyster mushrooms) provide riboflavin, niacin, potassium, and selenium, while contributing negligible cholesterol and fat. Valverde et al. (2015) emphasized mushrooms as sources of antioxidants, including polyphenols, ergothioneine, and vitamin C, which contribute to reducing oxidative stress. Ergothioneine, highlighted by Zhang et al. (2025), is now considered a conditionally essential micronutrient due to its role in protecting cells from oxidative damage.

β‐glucans, present in shiitake, *Agaricus blazei*, and oyster mushrooms, enhance immune modulation by interacting with dectin‐1 receptors, as discussed by Cerletti et al. (2021). This positions mushrooms as “immuno‐active foods.” Another functional classification arises from vitamin D_2_ enrichment. Cardwell et al. (2018) and Taofiq et al. (2017) reported that UV‐exposed mushrooms, such as shiitake and button mushrooms, convert ergosterol to vitamin D_2_, increasing serum 25(OH)D levels in human trials. This makes them one of the only non‐animal dietary vitamin D sources.

### 6.2 Production of Mushrooms

Global mushroom production is concentrated in a few genera. Royse et al. (2017) reported that *Lentinula* contributes ~22% of world output, followed by *Pleurotus* (~19%), *Auricularia* (~18%), *Agaricus* (~15%), and *Flammulina* (~11%). Carrasco et al. (2018) divided cultivation systems into compost‐based production (mainly *Agaricus*) and lignocellulosic “bag‐log” systems (used for *Lentinula*, *Pleurotus*, and *Auricularia*). Grimm and Wösten (2018) further positioned mushroom farming as a circular bioeconomy practice, utilizing agricultural residues to produce protein and bioactive compounds. FAO data analyzed by Wösten et al. (2020) showed a 30‐fold increase in mushroom production between 1978 and 2018, driven by demand for sustainable protein alternatives.

### 6.3 Responses of Mushrooms to Nanomaterials

#### 6.3.1 Growth Enhancers Using Nanoparticles

Kumari et al. (2025) validated that the use of zinc oxide nanoparticles (ZnO‐NPs) with Pleurotus substrates increased mycelial growth, promoted pinhead formation, and increased antioxidant enzyme activity. But beyond a certain level of nanoparticle use, this showed inhibitory effects. A nitrogen nano‐supplement (nano‐urea) was tested for its efficacy for yield enhancement in Pleurotus ostreatus and amino acid composition by Sassine et al. (2021), where the time of application played a significant role for positive results.

#### 6.3.2 Nanoparticles as Biofortifiers

Wang et al. (2025) found that selenium nanoparticles (Se‐NPs) reduced cadmium content in Agaricus blazei fruiting bodies but resulted in a higher proportion of organically‐bound selenium compounds such as selenomethionine (SeMet). This finding was supported by Madaan et al. (2024a, 2024b), which identified that selenium and zinc nanoparticles have the potential to increase micronutrient content in mushrooms and may be considered for use in biofortified functional foods.

#### 6.3.3 Nanotechnology in Postharvest Preservation

Sami et al. (2021b) had shown that edible coating blends of chitosan with nano‐silica and nisin were effective in slowing down browning and microbial deterioration of *Agaricus bisporus*. This prolonged shelf life beyond 10 days when initially only 5 days of shelf life was possible. In another study, Sami et al. (2020) had shown its efficacy.

#### 6.3.4 Risks and Considerations

Ameen et al. (2021a, 2021b) indicated a risk of interference with beneficial fungi in soil by metal nanoparticles such as silver nanoparticles (AgNPs), while Tian et al. (2019a, 2019b) pointed out harmful effects of nanoparticles toward beneficial fungal consortia. As such, using highly biocidal nanomaterials for preharvest purposes only but not for making substrates would be appropriate. Clear policies must be considered: The European Commission (2022) has amended its definition of nanomaterials. A minimum 50% presence in the 1 to 100 nm particle‐size range has been made mandatory. This conforms to standards for terminology prepared by ISO (2015).

#### 6.3.5 Comparative Performance of Different Nanomaterials in Mushroom Cultivation

A comparative assessment of different nanomaterials reveals important differences in their functional roles and mechanisms of action in mushroom cultivation systems. Metal and metal oxide nanoparticles such as zinc oxide (ZnO), iron oxide (Fe_2_O_3_), and titanium dioxide (TiO_2_) are frequently investigated for their ability to enhance nutrient availability and stimulate mycelial growth (Ngwenya et al. 2025). ZnO nanoparticles, for instance, have been reported to improve enzyme activity and micronutrient uptake, leading to increased yield and improved nutritional profiles in several edible mushroom species. In contrast, silver nanoparticles (AgNPs) are primarily explored for their strong antimicrobial properties, which can suppress pathogenic fungi and bacteria during both cultivation and postharvest storage (Kaya, C., et al. 2023). These nanoparticles contribute to improved crop protection and shelf‐life extension but may raise concerns related to potential toxicity at higher concentrations.

Carbon‐based nanomaterials such as graphene oxide and carbon nanotubes have also demonstrated growth‐promoting effects in some studies, possibly through enhanced water retention and improved nutrient transport within the substrate matrix. However, the effects of these materials appear to be highly dose‐dependent and species‐specific (Panaitescu, D. M., et al. 2024). These findings highlight that the selection of appropriate nanomaterials should be guided by specific cultivation objectives, such as yield optimization, biofortification, or postharvest preservation. Moreover, differences in experimental conditions, nanoparticle size, concentration, and application methods contribute to variability in reported outcomes, emphasizing the need for standardized protocols and comparative studies.

Mushrooms are classified as nutrient‐dense foods, immunoactive β‐glucans, ergothioneine antioxidants, and non‐animal vitamin D2 producers (Panda, S., et al. 2025). Their production is dominated by a few species and relies on compost and lignocellulosic systems. Nanomaterials offer promising roles as growth stimulants, biofortifiers, and postharvest preservatives, as shown in Table 5. However, they must be applied with caution due to potential toxicity and regulatory concerns. Future research should focus on optimizing dosage, long‐term safety, and integration with sustainability goals.

**TABLE 5:** Response of edible fungi to nanomaterials, active ingredients analyzed, and methods used.

Mushroom species/group	Nanomaterial type and mode of application	Physiological/food‐related response	Active ingredients assessed	Analytical methods used	References
*Pleurotus pulmonarius*	Biogenic ZnO nanoparticles (substrate amendment, 20–40 mg·kg^−1^)	Faster mycelial growth, early pinning, ↑ yield, ↑ antioxidant enzymes	Phenolics, flavonoids, antioxidant enzymes (SOD, CAT, POD), proximate nutrients	Spectrophotometry (Folin–Ciocalteu for phenolics; enzyme activity assays)	Kumari et al. (2025)
*Pleurotus ostreatus*	Nano‐urea nitrogen supplements (substrate spray or mix)	Modulated cycle time, ↑ yield, improved amino acid profile	Amino acids, protein, proximate composition	Amino acid analyzer (HPLC post‐column derivatization), proximate AOAC methods	Sassine et al. (2021)
*Agaricus blazei*	Selenium nanoparticles (substrate fortification, co‐contaminated with Cd)	↓ Cd uptake in fruiting bodies, ↑ organic Se forms (SeMet, SeCys), improved antioxidant defense	Selenium species, Cd content, antioxidant enzymes	HPLC‐ICP‐MS (Se speciation), AAS/ICP‐OES (Cd, total Se), enzyme assays	Wang et al. (2025)
*Agaricus bisporus*	Nano‐silica + chitosan + nisin edible coatings (postharvest)	↓ Browning, ↓ microbial spoilage, extended shelf life (10–12 days vs. 5)	Polyphenol oxidase, peroxidase, total phenolics, microbial load	Enzyme activity assays, microbial plating, Folin–Ciocalteu	Sami et al. (2021b)
*Agaricus bisporus*	TiO_2_ nanocomposite coating (postharvest)	Reduced respiration rate, delayed senescence, better firmness	Respiration rate, antioxidant activity, proximate composition	Gas chromatography (respiration gases), DPPH/RSA assays, proximate AOAC	Sami et al. (2020)
*Pleurotus* spp.	Green‐synthesized ZnO‐NPs (leaf extract‐mediated)	Enhanced yield, enriched bioactive profile, ↑ polysaccharides	β‐glucans, phenolics, polysaccharides	Phenol‐sulfuric acid assay, HPLC for sugars, UV–Vis spectrophotometry	Wang et al. (2024b)
General edible fungi	Ag nanoparticles (substrate or coatings)	Antimicrobial protection, ↓ competitor fungi, potential toxicity at high dose	Residual Ag, microbiome diversity	ICP‐MS (Ag quantification), 16S/ITS sequencing for microbiota	Ameen et al. (2021a, 2021b)

### 6.4 Response of Edible Fungi to Nanomaterials and Nano‐Bio Stimulants

Recent studies have highlighted the potential role of nanomaterials (NMs) and nano‐biostimulants in enhancing fungal growth, stress resistance, and metabolite production in edible fungi. Responses of edible fungi; (i) Growth stimulation: Low concentrations of metallic nanoparticles (Ag, ZnO, TiO_2_) and carbon‐based nanomaterials have been reported to improve mycelial growth and fruiting body yield in *Pleurotus* and *Ganoderma* species (Abd‐Elsalam 2024; Nazim et al. 2024). (ii) Stress tolerance: Nano‐silicon and nano‐chitosan enhance fungal tolerance to abiotic stress (e.g., oxidative imbalance, salinity) by activating antioxidant enzyme systems (Mridha et al. 2025; Wei et al. 2025). (iii) Secondary metabolites: Nano‐biostimulants such as nanoclay, nanohydroxyapatite, and carbon quantum dots promote bioactive metabolite accumulation (flavonoids, polysaccharides) important for medicinal and nutritional value (Andleeb et al. 2025; Miguel‐Rojas et al. 2023). (iv) Toxicity concerns: Excessive nanoparticle exposure (especially AgNPs and CuO NPs) can cause mycelial inhibition or induce ROS‐mediated damage, suggesting a dose‐dependent response (Islam et al. 2025; Wang et al. 2024a). As shown in the Table 6.

**TABLE 6:** Response of edible fungi (mushrooms) to nanomaterials and catalysts as sustainable healthy food.

Nanomaterial/catalyst	Mushroom species	Observed fungal response	Enhanced nutrients/bioactive compounds	Analytical methods	References
Magnetic iron nanoparticles (FeO NPs)	*Agaricus bisporus*	Boosted metabolic activity; improved fruiting body biomass	↑ Vitamin D, B2, B6	HPLC; UV–Vis	Algarawi et al. (2025a)
Carbon nanotubes (CNTs)	*Agaricus bisporus*	Improved nutrient uptake; stimulation of enzymatic activity	↑ B‐complex vitamins; antioxidants	HPLC; LC–MS/MS; GC–MS	Algarawi et al. (2025b); Rajput et al. (2021)
Silver nanoparticles (AgNPs)	*Pleurotus ostreatus*	Antimicrobial effect in substrate; improved fungal resistance	↑ Phenolics; flavonoids	FTIR; DPPH antioxidant assay	Vetchinkina et al. (2017)
Zinc oxide nanoparticles (ZnO NPs)	*Ganoderma lucidum*; *Pleurotus* spp.	Stimulated mycelial growth; pathogen resistance	↑ Ergothioneine; polysaccharides	HPLC; NMR	Ahmed et al. (2022)
Titanium dioxide nanoparticles (TiO_2_ NPs)	*Agaricus bisporus*	Light‐activated catalysis; enhanced vitamin D2 under UV	↑ Vitamin D2	HPLC; UV–Vis	He et al. (2020)
Copper nanoparticles (Cu NPs)	*Pleurotus florida*; *Ganoderma lucidum*	Enhanced laccase enzyme activity; growth stimulation	↑ Polyphenols; antioxidant activity	Enzyme assays; LC–MS	Sharma et al. (2019)
Gold nanoparticles (Au NPs)	*Ganoderma lucidum*	Biocompatible stimulation of metabolism	↑ Secondary metabolites (alkaloids, terpenoids)	LC–MS/MS; HPLC	Singh et al. (2020)
Silicon nanoparticles (Si NPs)	*Pleurotus ostreatus*	Strengthened fungal cell walls; stress tolerance	↑ β‐glucans; chitin	FTIR; NMR	Yadav et al. (2021)
Chitosan nanoparticles	*Agaricus bisporus*; *Pleurotus* spp.	Elicitor of defense pathways; improved immunity	↑ β‐glucans; phenolic content	HPLC; Folin–Ciocalteu assay	Malerba and Cerana (2018)
Effective microorganisms (EM) + nanomaterials	*Agaricus bisporus*	Synergistic biosynthesis of vitamins; resilience to stress	↑ Vitamins A, E, B5; polysaccharides	HPLC; ELISA; LC–MS	Algarawi et al. (2025a)
Plant‐based biostimulants (e.g., Atonik, Seaweed extracts)	*Agaricus bisporus*; *Pleurotus ostreatus*	Enhanced enzymatic activity; promoted yield	↑ Vitamins A, C, E; polysaccharides	HPLC; GC–MS	Algarawi et al. (2025b); Xu and Smith (2022)
Enzyme catalysts (laccase, peroxidase, cellulase)	*Ganoderma lucidum*; *Pleurotus* spp.	Improved substrate degradation; nutrient release	↑ Phenolics; essential amino acids	Enzyme kinetics; HPLC	Baldrian (2006)

Methods used: (i) In vitro mycelial assays → measuring radial growth on NM‐amended media (Kalwani et al. 2022). (ii) Fruit body yield experiments → biomass, sporophore count, and morphological traits in cultivation trials (Mi et al. 2020; Zhao, H., et al. 2021). (iii) Biochemical assays → antioxidant enzyme activities (SOD, POD, CAT), lipid peroxidation, and metabolite profiling (Nazim et al. 2024; Mridha et al. 2025). (iv) Molecular approaches → qPCR for stress‐responsive genes and transcriptomic analysis of metabolic pathways (Miguel‐Rojas et al. 2023; Islam et al. 2025). (v) Microscopy and spectroscopy → SEM/TEM, FTIR, and XRD to detect NM uptake, localization, and interaction with fungal cell walls (Abd‐Elsalam 2024; Wang et al. 2024a). As shown in Table 6. Overall, edible fungi exhibit stimulatory or inhibitory responses depending on NM type, concentration, and exposure method. Controlled application of nano‐biostimulants offers potential in sustainable mushroom production, but requires standardized toxicity thresholds.

## 7 Challenges

Safety and toxicology: High‐dose NPs may be cytotoxic; residues in mushrooms could affect consumers and soil microbiota (Mkhize et al. 2022; Muzammil et al. 2023; Mgadi et al. 2024).

Environmental fate: NP accumulation in SMS may impact soil and aquatic ecosystems; chemical modifications alter toxicity (Elsakhawy et al. 2022).

Standardization and regulation: No dedicated regulatory framework exists for nano‐biostimulants in mushroom cultivation (European Union 2019).

Cost and scalability: Green NP synthesis may lack economic scalability (Prokisch et al. 2024).

Mechanistic knowledge gaps: NP uptake, transformation, and distribution mechanisms in mushrooms require further investigation (Anuța et al. 2025).

## 8 Future Prospects

Safe‐by‐design nanomaterials: Biodegradable carriers like chitosan reduce risks and enhance biostimulant efficacy (Camele et al. 2024). Circular bioeconomy: Mushroom‐derived materials (e.g., chitosan) support SDG 12 via reusable NP formulations (Elsakhawy et al. 2022). Precision farming: Integration of NPs with precision delivery enhances nutrient efficiency and disease management (Prokisch et al. 2024). Food security and nutrition: Nano‐biofortification with Zn and Se addresses micronutrient deficiencies while mitigating heavy metal accumulation (Ali et al. 2024; Luo et al. 2025a, 2025b). Collaborative research and policy: Interdisciplinary approaches are needed for safety standards and residue monitoring (Anuța et al. 2025; European Union 2019).

## 9 Research Gaps and Future Perspective

Although substantial progress has been made in understanding the role of nanomaterials in mushroom cultivation, several research gaps remain. First, most available studies have been conducted under controlled laboratory or small‐scale experimental conditions. Large‐scale field validation under commercial cultivation environments is still limited, making it difficult to evaluate the economic feasibility and long‐term sustainability of nano‐enabled mushroom production systems. Second, there is a lack of standardized protocols regarding nanoparticle dosage, application methods, and substrate interactions. Differences in nanoparticle size, concentration, surface properties, and experimental conditions contribute to inconsistent results across studies. Establishing standardized methodologies would significantly improve comparability and reproducibility. Third, the environmental fate and long‐term ecological impacts of nanoparticles used in mushroom cultivation remain poorly understood. In particular, the accumulation of nanomaterials in cultivation substrates and their subsequent transfer to soil ecosystems requires further investigation. Finally, comprehensive life‐cycle assessments and sustainability evaluations are still scarce. Future research should integrate environmental, economic, and social dimensions to determine whether nanotechnology can genuinely contribute to sustainable and resilient mushroom production systems.

## 10 Conclusions

Nanotechnology is emerging as a promising tool for improving productivity, nutritional quality, and postharvest stability in edible mushroom production systems. The unique physicochemical properties of nanomaterials enable enhanced nutrient delivery, improved pathogen control, and the development of advanced preservation technologies. These innovations have the potential to support sustainable agriculture by increasing resource‐use efficiency and reducing food losses along the supply chain. Furthermore, the integration of nanotechnology with circular bioeconomy approaches, including the valorization of spent mushroom substrates and myco‐mediated nanoparticle synthesis, offers new opportunities for environmentally sustainable production systems. Such approaches contribute to broader sustainability goals by promoting waste valorization, resource recovery, and climate‐resilient food production. Nevertheless, the successful implementation of nanotechnology in mushroom cultivation requires careful consideration of safety, environmental impacts, and regulatory frameworks. Future research should focus on large‐scale validation studies, standardized application protocols, and comprehensive risk assessments to ensure that nano‐enabled mushroom production systems are both effective and sustainable.

## Author Contributions

**Fanar Hashum Al‐Hashemi:** supervision, investigation, writing – original draft, validation. **Hassanein H. Al‐juthery:** investigation, formal analysis, software, validation, resources, supervision. **Heidar Meftahizade:** investigation, visualization, data curation, supervision, writing – review and editing. **Hayyawi W. A. Al‐juthery:** conceptualization, validation, project administration. **Rukaibaa A. Chechan:** conceptualization, validation, project administration, data curation.

## Funding

The authors have nothing to report.

## Ethics Statement

The authors have nothing to report.

## Consent

The authors have nothing to report.

## Conflicts of Interest

The authors declare no conflicts of interest.

## Data Availability

The data that support the findings of this study are available from the corresponding author upon reasonable request.
